# Dr. Lubbock, on Cow-Pock

**Published:** 1801-01

**Authors:** Richard Lubbock

**Affiliations:** Norwich


### *To the Editors of the Medical and Phyfical Journal.*

Gentlemen,

FROM the communications upon the Cow-pock, a fubject intimately connected with the interefts of humanity, and defervedly exciting the attention of the medical world, I think the Medical Journal has been of the greateft utility; and it was with the greateft pleafure that, in the laft number of your Journal, I read the remarks of Sir Chriftopher Pegge of Oxford, and of Dr. Barry of Cork, upon the fame fubject. It appears from Sir C. Pegge's obfervations, that horfes are liable, in their heels, to the fame difeafe as affects the teats of cows, cows; from which fact, the fuggeftion of Dr. Jenner, concerning the occafional origin of the difeafe, would feem to be eftablifhed; and perhaps further obfervations may fhew, that other animals are not exempt from the action of this morbific matter. And Dr. Barry has rendered it probable from traditional teftimony, that a difeafe affecting cows, in a fimilar manner to the Cow-pock, and from them communicable to the human fubject, has been known in Ireland for a long time; it would feem alfo, that this affection was more general and frequent, in former times, than at prefent.

Among the Irifh, as we are told by Dr. Barry, this difeafe is known by the name of Shinach; a term, I prefume, of Celtic extraction, and belonging to the Galic language. And feeling the importance of etymological inquiries, when cautioufly purfued, in illuftrating the manners, cuftoms and opinions of antiquity, and in reviving the primary fignification of terms, I was induced from curiofity, and without any pretenfion to a knowledge of the Galic language, to look over in a curfory manner, fome collections and remains of that Celtic dialect, which I found among my other books; and I was much furprifed and gratified in obferving, that it is very probable the word SHINACH is a compound Galic term expreffive of a Cow's Teat; for from thefe collections I find, that the word SINNE, in Galic, means a teat, and the word AGH, in the fame language, is a common term for a cow: So that it would feem, that SHINACH was originally written SINNEAGH; whence it will be readily admitted, by every perfon in any degree acquainted with the converfions and changes taking place in the hiftory and progrefs of language, that in fubfequent periods, the compound term SINNEAGH was varioufly pronounced and written, as Sinnagh, Sinnach, and Shinach. Similar inftances to the contraction of Sinne into Sin, in the firft member of the compound, are fo frequent in all languages, that it is unneceffary to cite examples of it; and the converfion of the foft guttural confonant G, in the laft member, into the hard guttural confonant C, as well as the infertion of the afpirate H, in the firft fyllable, are changes fo familiar and conformable to the received principles of etymology, that I fhall dwell no longer upon them.

Thefe remarks, although avowedly more literary than practical, feem to me of no inconfiderable importance, as they fhew, if juft, that the knowledge of the exiftence of the Cow-pock has been coeval with the language of the very early fettlers in Ireland; and it would appear from them, that among the ancient Irifh, in remote antiquity, this difeafe was not only well underftood to affect cows, but that its influence over the human man body was known and eftablifhed, as the name given it, when exhibiting itfelf in the human fubject, was the term in the ancient language, characterifing the part of the animal whence the difeafe was derived; fo that a perfon having this difeafe was faid to have the Shinach or Cow's Teat. And this mode of transferring the names of caufes to their effects being the moft natural, is the moft eafy procefs of the human mind in the advancement of knowledge. Many terms of every fcience may ferve as documents of this kind of nomenclature; but to confine ourfelves to fimilar inftances of difeafes, named either immediately from their caufes, and from fome refemblance to things external, or the effects of things external to the body, we need only turn over the common fyftems of nofology; when we fhall find Raphania, a difeafe named from its fuppofed connection with a plant as its caufe. Frambœfia, another difeafe, named from its likenefs to a fruit. Urticaria from its fimilarity to the effects of a nettle, and Elephantiafis from fome refemblance, either to the natural form, or to fome difeafed ftate of the members of the elephant.

Thefe remarks, which may feem fanciful to fome, I fhall not extend any farther, but fhall leave them to be confirmed or confuted by perfons having greater opportunities of inveftigating their truth, from a greater knowledge of the Celtic language and its dialects, or from a more frequent intercourfe with perfons ufing that language. I am,

Gentlemen,

Your obedient fervant,

*Norwich, Nov.* 23, 1800. RICHARD LUBBOCK.

